# Brain delivery of quercetin-loaded exosomes improved cognitive function in AD mice by inhibiting phosphorylated tau-mediated neurofibrillary tangles

**DOI:** 10.1080/10717544.2020.1762262

**Published:** 2020-05-13

**Authors:** Yao Qi, Lin Guo, Yibing Jiang, Yijie Shi, Haijuan Sui, Liang Zhao

**Affiliations:** a School of Pharmacy, Jinzhou Medical University, Jinzhou, P R China;; b Department of Pharmacology, Jinzhou Medical University, Jinzhou, P R China

**Keywords:** Quercetin, Alzheimer’s disease, exosomes, bioavailability, Tau

## Abstract

It is reported that quercetin (Que) can prevent tau pathology and induce neuroprotection by improving cognitive and functional symptoms in the treatment of Alzheimer’s disease (AD). However, its clinical application has been limited due to its poor brain targeting and bioavailability. Exosomes are considered as cargo carriers for intercellular communication and especially serve as a natural and important drug brain delivery platform for achieving better treatment of central neurological diseases. Here, we developed plasma exosomes (Exo) loaded with Que (Exo-Que) to improve the drug bioavailability, enhance the brain targeting of Que and potently ameliorate cognitive dysfunction in okadaic acid (OA)-induced AD mice. Our results showed that Exo-Que improved brain targeting of Que as well as significantly enhanced bioavailability of Que. Furthermore, compared with free Que, Exo-Que better relieved the symptoms of AD by inhibiting cyclin-dependent kinase 5 (CDK5)-mediated phosphorylation of Tau and reducing formation of insoluble neurofibrillary tangles (NFTs), suggesting its therapeutic potential for better treatment of AD.

## Introduction

Alzheimer’s disease (AD) is the most common type of progressive neurodegenerative diseases associated with learning and memory deficits caused by neurological dysfunction (Lane et al., 2018). Clinically, it is characterized by dementia such as memory impairment, executive dysfunction, and behavioral change. Some potential mechanisms have been proposed to explain the underlying pathology of AD including formation of senile plaques induced by amyloid deposition, tau protein hyperphosphorylation and formation of insoluble neurofibrillary tangles (NFTs) (Gao et al., 2018). Nowadays, the available clinical option of medication therapies for enhancing cognitive and functional symptoms is very limited and mainly includes some cholinesterase inhibitors and memantine (Epperly et al., 2017). Although these drugs have been shown to alleviate functional decline in some patients, they fail to halt the pathological progression from mild to severe AD. Therefore, developing suitable and alternative medicines to achieve effective pharmacologic AD therapy is of great value.

Cyclin-dependent kinase 5 (CDK5) as a unique member of the cyclin-dependent kinase families plays an important role on regulating pathophysiological features in AD pathogenesis (Lu et al., 2020). When AD occurs, the activity of CDK5 in neuron becomes abnormally active, inducing abnormal tau hyperphosphorylation and accelerating their aggregation into filaments or tangles, eventually leading to synaptic loss and neuronal death (Shen et al., 2018). Some drugs are reported to downregulate CDK5 in AD mice and abrogate Tau-associated neurological disorders by inhibiting Tau hyperphosphorylation (Das et al., 2019; Zeb et al., 2019). This mechanism provides us to find an effective drug to inhibit CDK5-mediated phosphorylation of Tau, thereby alleviating and even curing AD.

Quercetin (Que) as a flavonoid natural compound has been recognized as a promising cognitive enhancer owing to its potential pharmacological effects including neuroprotection, anti-oxidation, and anti-inflammation (Khan et al., 2018). Especially, it was reported that Que can prevent tau pathology, inhibit amyloid production and induce neuroprotection associated with autophagy (Kuo et al., 2019). However, its poor solubility, low bioavailability and difficulty in crossing the brain, impeded clinical development of Que as a potential therapeutic agent (Vinayak & Maurya, 2019).

For most therapeutic agents like Que for AD therapy, existence of blood–brain barrier (BBB) remains a large obstacle to improving drug therapeutic efficacy for the treatment of AD (Zhou et al., 2019; Ramalho et al., 2020). Owing to BBB unique structure such as tight junctions between endothelial cells, astroyctic endfeet and a basement membrane, BBB as a self-protective defendence isolates the brain from harmful blood-borne substances and microorganisms (Zenaro et al., 2017; Yamazaki & Kanekiyo, 2017). Similarly, it also prevents the drug from crossing the BBB when administered peripherally. Almost all drugs with high molecular weight and more than 98% of low molecular weight drugs cannot pass through BBB, thus significantly reducing their therapeutic efficacy in brain (Elias et al., 2001; Pardridge, 2005; Re et al., 2012; Maussang et al., 2016). In order to enhance the accumulation of drug in brain, a tremendous dose of drugs have to be applied *in vivo*, thus posing the potential risk of systemic toxicity and severe adverse effects. Therefore, it is critical to find a novel approach aiming at enhancing simultaneous BBB-crossing ability of drugs for treating AD and improving neurological outcomes.

Exosomes as nano-size vesicles secreted by living cells hold a promising potential as a drug delivery carrier in charge of transporting drugs into the specific sites or organs. Compared with other inorganic and organic cargo carriers, exosomes possess many advantages over good compatibility, low immunogenicity, innate stability and high transmission efficiency, so they are widely used as delivery tools for packing proteins, nucleic acids and chemicals in clinical area (Lener et al., 2015; Fais et al., 2016). However, naïve exosomes depend on its inherited nature to passively target and accumulate some specific organs like liver and spleen, thus reducing its targeting efficiency in other organs and weakening drug therapeutic efficacy in disease treatment, especially in central nervous disease therapy. Nowadays, therapeutic exosomes were modified by specific recognizable ligands and achieved drug targeted delivery. Researchers have found that some aptamers used as targeting agents, could be modified onto the surface of exosomes and achieved active targeting therapy with high specificity, selectivity, and affinity (Tian et al., 2018; Zou et al., 2019; Luo et al., 2019).

Plasma derived exosomes (Exo) are 40–150 nm nanosized extracellular vesicles found in blood plasma and contain complex RNA and proteins (Sundar et al., 2019; Cumba Garcia et al., 2019). They possess the unique properties including the innate ability of crossing the BBB, immunologic inertia and stability (Blanc et al., 2005). Especially, a large number of studies have shown that Exo as a promising carrier not only improves the bioavailability of drugs but also achieves drug brain targeted delivery across BBB. In addition, some peptides inherited by Exo can specifically bind to receptors in the brain, thereby accelerating the accumulation of Exo in the brain (Mulvihill et al., 2020).

**Figure 1.**
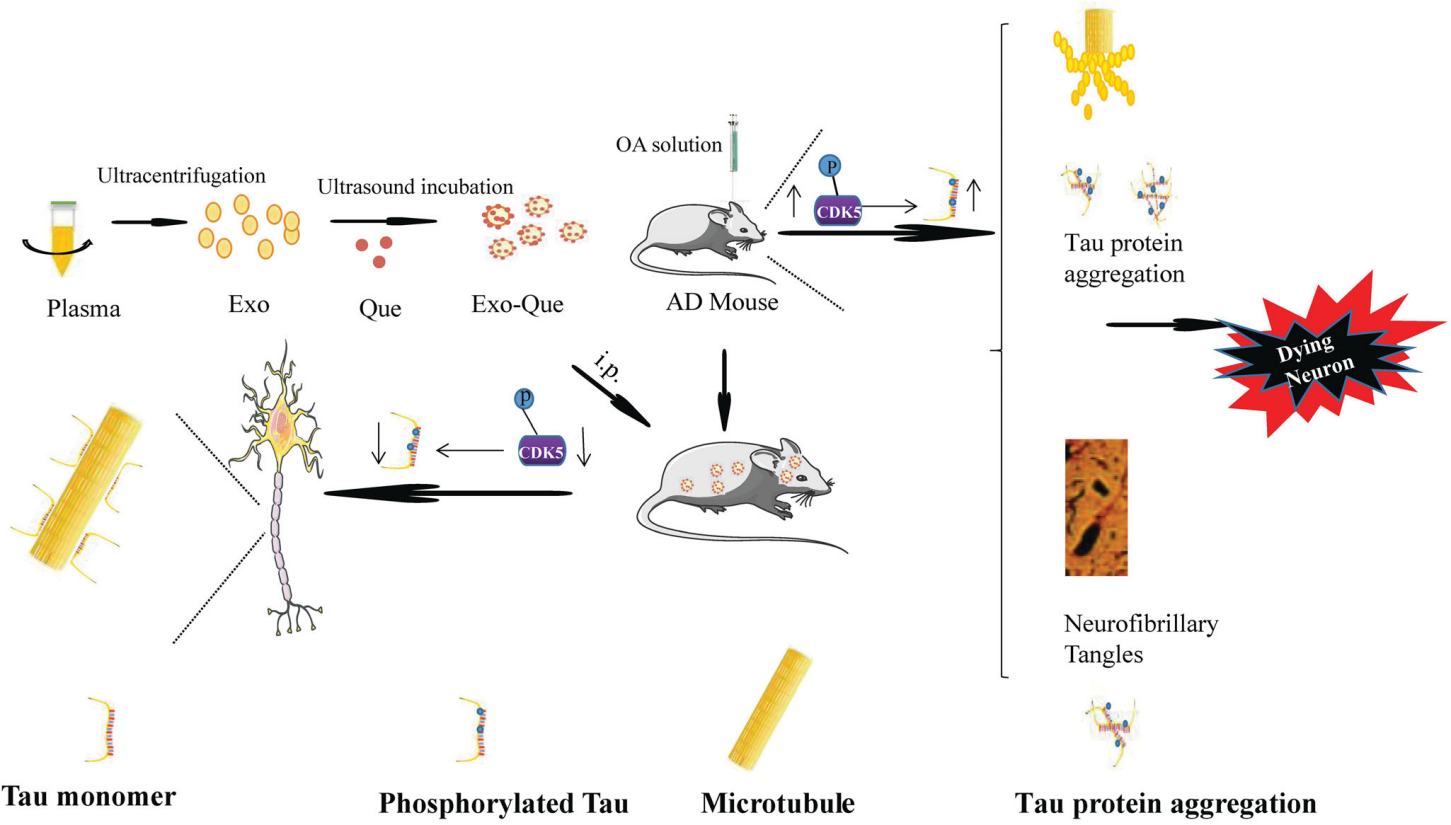
Primary hypothesis of this study. Exo-Que enhanced the bioavailability and achieved brain targeting *in vivo*. Exo-Que improved cognitive and functional symptoms in OA-induced AD mice by inhibiting CDK5-mediated phosphorylation of Tau and reducing NFTs.

Based on the previous report on treatment of AD with Que from the perspective of the regulation of tau phosphorylation and inherited ability of Exo on targeting brain, we aimed at fabricating plasma derived Exo packed with quercetin (Exo-Que) to enhance drug bioavailability and achieve brain targeting *in vivo*. Okadaic acid (OA) was used to establish an animal model of AD with high Tau hyperphosphorylation (Xu et al., 2018). We investigated effect of Exo-Que on inhibiting CDK5-mediated Tau phosphorylation and reducing the formation of NFTs. Furthermore, the beneficial role of Exo-Que on attenuating OA-induced learning and memory deficits was further evaluated in OA-induced AD mice. We hypothesized that Exo-Que contributed to its neuroprotective effects in OA-induced AD mice by improving brain targeting of Que and inhibiting CDK5-mediated Tau phosphorylation (Figure 1).

## Materials and methods

### Materials

Que was purchased from Aladdin Chemical (Shanghai, China). The primary antibodies used in western blot analyses against CDK5, p-CDK5, Tau, p-Tau, cleaved caspase 3, cleaved caspase 9 and the secondary antibody goat anti-rabbit IgG/HRP were all obtained from Abcam (Cambridge, UK). Other chemicals purchased were of analytical grade and obtained from Sigma-Aldrich.

### Exo-Que isolation and characterization

According to the previous reports (Wang et al., 2019; Xiong et al., 2020), in order to obtain Exo-Que, whole blood of SD rats at age 7–8 weeks supplied by Jinzhou Medical University Animal Center was collected and treated with 0.1% sodium heparin. The exosome pellets were isolated and obtained from blood plasma by the gradient centrifugation in a Type CS150GXII ultracentrifuge (Hitachi, Koki, Co., Ltd, Japan), and analyzed using a BCA Protein Assay kit (Beyotime, Shanghai, China) (Qu et al., 2018). The encapsulation of Que in Exo was performed as follows: 1 mg Que was weighed and dissolved in 200 μl DMSO containing 2% Tween-80, and then added with 1.5 mg Exo under continuous shaking at 4 °C overnight, followed by continuous ultrasonic incubation in an ice-water bath. The obtained Exo-Que was washed three times for removing the unencapsulated Que and Que in supernatant was obtained and measured by high performance liquid chromatography (HPLC). The chromatographic system included a Hypersil C18 column, a mobile phase containing methanol and phosphoric acid in water (pH 7.0; 0.01 M) (60:40, v/v), at a flow rate of 1.0 mL/min, column temperature at 30 °C and the UV detection wavelength was set at 374 nm. Encapsulation efficiency (EE%) and loading rate (LR%) of Que were calculated according to the previous report (Su et al., 2014). The morphology and shape of Exo and Exo-Que were investigated using an atomic force microscopy (AFM, FM-Nanoview6800; FSM-PRECISION, Suzhou, China). Particle size of Exo and Exo-Que were measured by Zetasizer Nano ZS; (Malvern Instruments, Malvern, UK). Western blotting was used to detect the expression levels of the exosomal marker proteins such as CD63 and Alix.

### Pharmacokinetic studies in SD rats

According to our previous publication (Wang et al., 2019), pharmacokinetic profile and bioavailability of Que and Exo-Que were investigated as follows: when a single dose of various Que formulations at 12 mg/kg was intravenous administrated in SD rats which aged at 7–8 weeks and weighed 200 ± 18 g from Jinzhou Medical University Animal Center. Blood samples of rats were collected periodically and then centrifuged to obtain plasma. After precipitating the protein in the plasma by hydrochloric acid and ethyl acetate deposition, the supernatant was collected and the content of Que in the supernatant was determined by HPLC. According to the concentration change of Que in the blood over time, blood drug concentration–time curves in Que group and Exo-Que group were obtained. After 1 h of intravenous administration of Que and Exo-Que, all animals were euthanized and brain tissues were collected rapidly. Finally, the concentration of Que in brain was determined by HPLC.

### Morris water maze test

**Figure 2.**
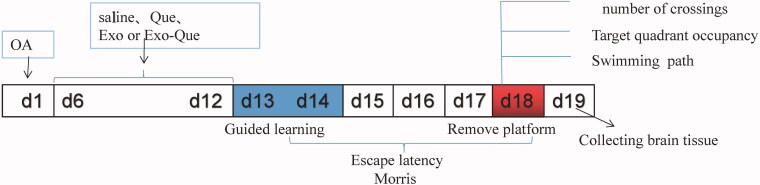
Experimental design for the animal study.

According to our previous reports, the animal model of AD was established by injecting 2 μL of OA (0.2 μM; Upstate Biotechnology, Inc., Lake Placid, NY, USA) into dorsal hippocampal area of C57BL/6 mice from Jinzhou Medical University Animal Center once per day for 5 consecutive days and experimental design was shown in Figure 2. Naïve Exo, free Que and Exo-Que (0.6 mg of Que per mL, 200 μL/day) were administered by peritoneal injection once per day for consecutive 7 days. After that, Morris Water Maze test was applied to evaluate spatial learning and memory of mice. The experimental protocol accorded with the National Guidelines for Animal Protection and was proved by the Institutional Animal Care and Use Committee of Jinzhou Medical University.

### H&E staining

Paraffin sections of brain were dewaxed in water followed by continuous incubation with hematoxylin. After 5 min, sections were treated with 1% hydrochloric acid alcohol for color separation, and were stained with eosin for 3 min. When being dehydrated in gradient alcohol and transparented using xylene, sections were sealed with neutral gum and observed under a microscope.

### Immunofluorescence staining

According to our previous report (Zheng et al., 2019), we performed immunofluorescence staining as follows: the isolated brains were cryosectioned at 15 μm thickness. After treated with Triton-100 (0.1%, 25 min) and serum (10%, 1 h) at 37 °C, and then immunostained with antibodies overnight at 4 °C. Following PBS washing for 3 times, the samples were incubated with fluorophore conjugated secondary antibodies (Invitrogen, Grand Island, NY, USA) for 1 h at room time. Next, the tissue slides were further washed 3 times with PBS. After mounting with ProLong Antifade Reagents (Invitrogen), the tissue slides were imaged using an FV10i confocal microscope (Olympus, Tokyo, Japan). In all the staining series PBS was used instead of primary antibodies as negative controls. Images were processed and analyzed by ImageJ software (NIH). All settings of imaging and processing were kept constant, and the relative fluorescence intensities were calculated.

### Western blotting analysis

According to our previous report (Zheng et al., 2019), after drug treatment, proteins in the hippocampus of brain tissue were extracted and assayed by western blotting. The following antibodies were used: anti-CDK5 (1:500), anti-phosphor-CDK5 (pTyr15) (1:1000), anti-tau (1:500), anti-phosphor-tau (T231) (1:5000), anti-cleaved caspase3 (1:500), anti-cleaved caspase9 (1:1000) and β-actin (1:1000). Finally, the targeted proteins were visualized and analyzed using gel analysis system.

### Silver staining

According to the previous report (Aboud & Griffin, 2014), silver staining was performed as follows: after being deparaffinized and hydrated, paraffin sections were treated with 20% silver nitrate in 60 °C for 15 min. With the double washing with distilled water, sections were subsequently incubated with ammoniacal silver solution (15 min) and sodium thiosulfate solution (2 min). The sections then were dehydrated and mounted with synthetic resin. The structure of NFTs in brain section was observed under a confocal microscope.

## Results

### Characterization of Exo and Exo-Que

**Figure 3.**
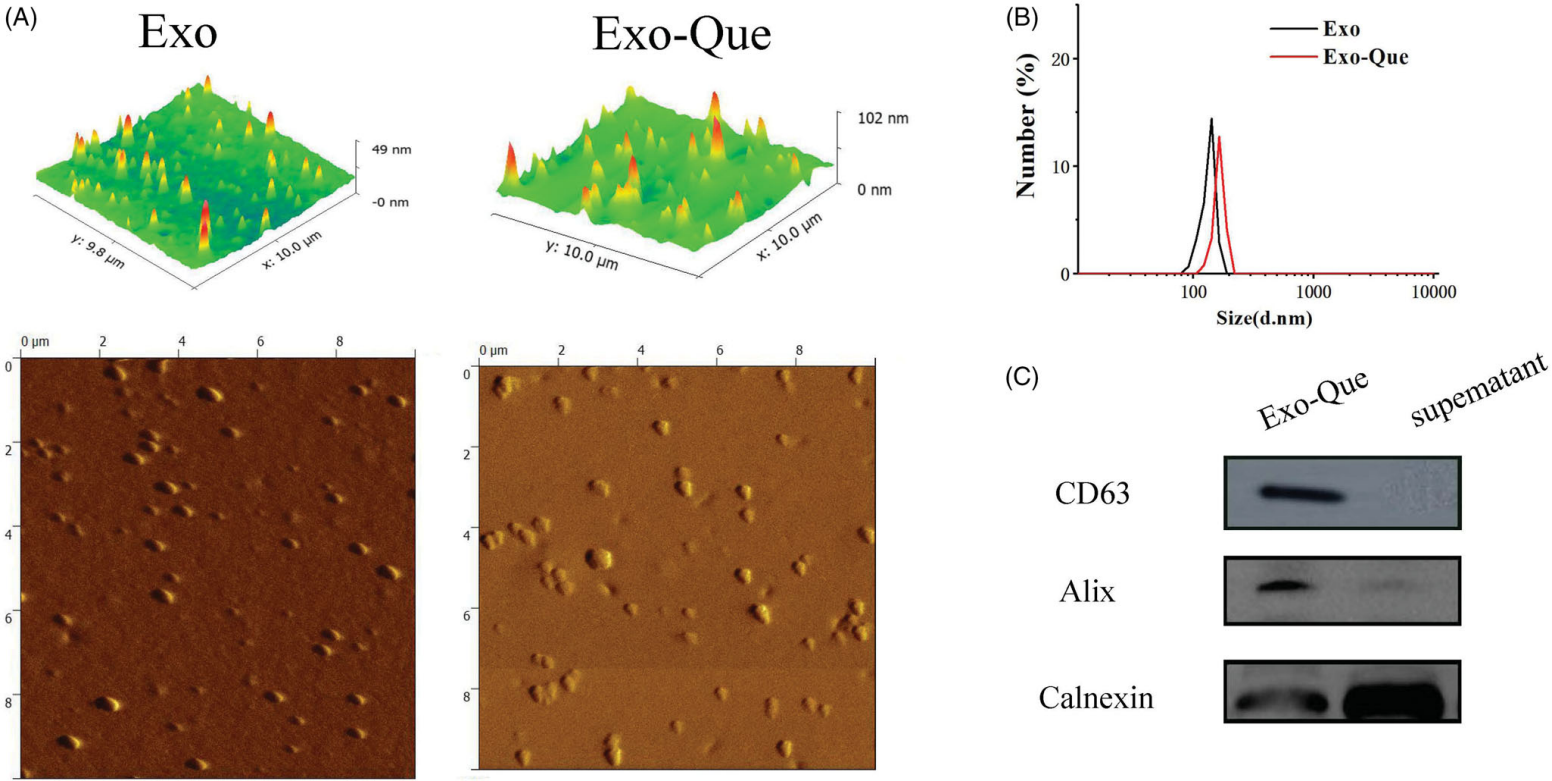
Characterization determination of Exo and Exo-Que. (A) Morphology of Exo and Exo-Que by AFM. (B) Particle size distribution of Exo and Exo-Que. (C) Detection of exosomal specific markers by western blot.

Purified Exo was collected by using a gradient ultracentrifugation and Que was encapsulated into Exo by ultrasonification as described in Materials and Methods. Approximately 33.3 μg purified Exo accounted at 2.5 × 10^9^ particles could be finally produced from 1 mL rats plasma. We first characterized the Exo and Exo-Que by observing their morphology and shape using AFM. The pictures captured using AFM showed particles with typical homogeneous and spherical vesicles, as published before (Figure 3(A)). Particle size of Exo and Exo-Que was measured by Zetasizer Nano ZS (Malvern Instruments, Malvern, UK). The diameters of Exo and Exo-Que were mostly distributed at 125 nm and 150 nm. Western blot analysis in Figure 3(A)(C) showed the presence of exosomal proteins such Alix and CD63 in Exo-Que. The above results inferred that vesicles we obtained by ultracentrifugation were basically Exo. There were no obvious differences on the particle size and protein component between Exo and Exo-Que, indicating that the properties of Exo were not affected by the loading of Que. We also obtained an encapsulation efficiency of Que at 30 ± 8.3% and drug loading ratio of Que at 17.3 ± 6.34%.

### The bioavailability of Que was enhanced via loading into Exo

**Figure 4.**
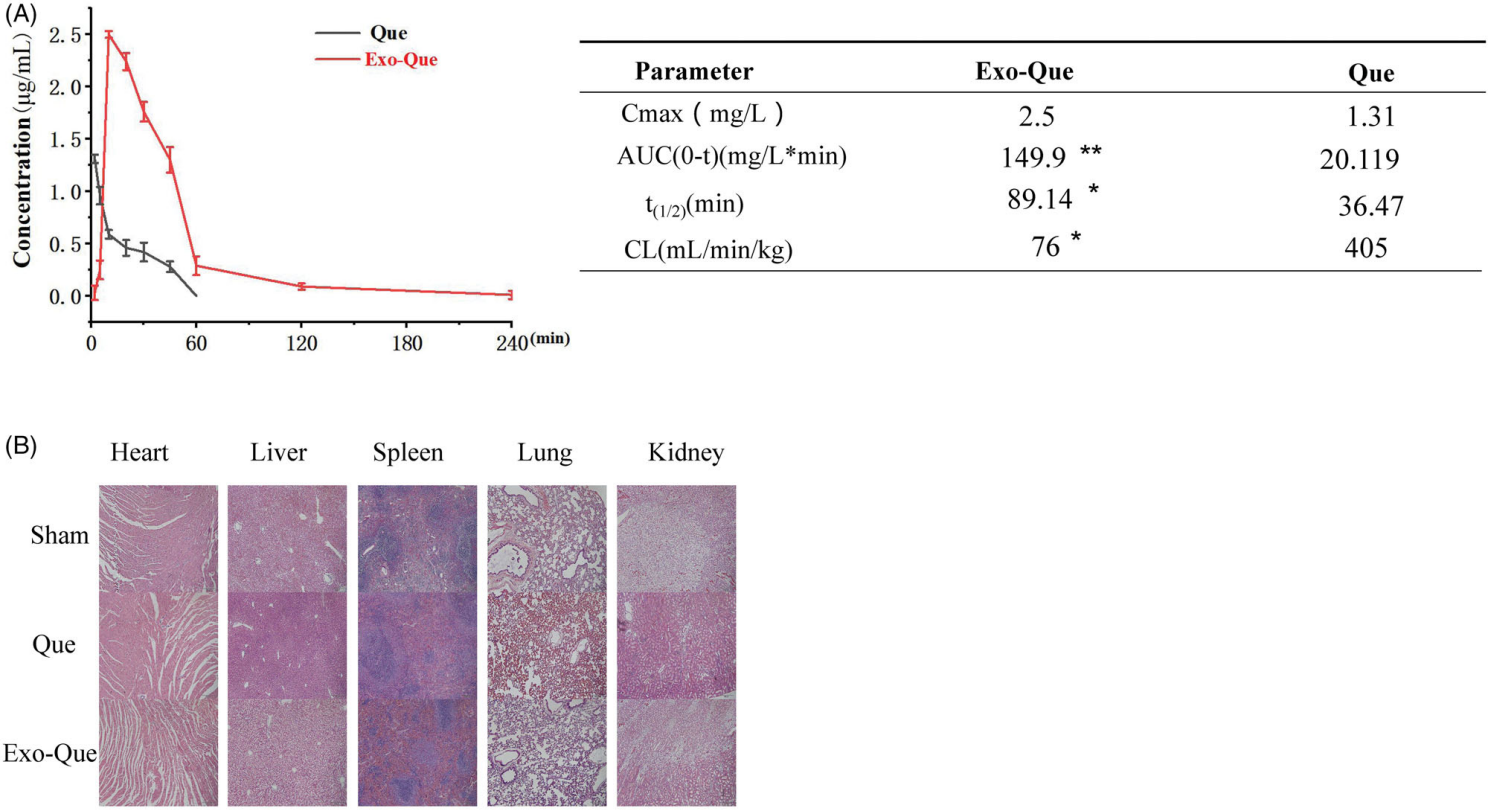
Exo-Que enhanced the bioavailability of Que *in vivo*. (A) The plasma concentration–time curve and fitted PK parameters of Que and Exo-Que after single i.v. administration of Que and Exo-Que at the concentration of Que (12 mg/kg). Data are expressed as means ± SD (*n* = 3). *p < 0.05 and **p < 0.01. (B) H&E staining of heart, liver, spleen, lung, and kidney tissue sections of rats at 7 day after i.v. daily administering a single dose of 1 mL of saline and 1 mL of various Que formulations at 12 mg/kg. The scale bar is 200 μm and applies to all figure parts.

In order to overcome the low bioavailability of Que, we aimed at encapsulating Que into Exo and evaluating its pharmacokinetic profiles by administering intravenously (i.v.) in SD rats. The pharmacokinetic parameters of free Que and exosomal Que were analyzed using DAS software and shown in Figure 4(A). The results revealed that when the Que and Exo-Que at the same concentrations of Que (12 mg/kg) were administrated via intravenous injection, exosomal Que significantly enhanced its pharmacokinetic profile as compared to free Que with prolonged half-life (t_1/2_) (89.14 min vs 36.47 min), higher maximum plasma concentration (Cmax, 2.5 mg/L vs 1.31 mg/L). The area under the plasma concentration–time curve from zero time to infinity (AUC_0-t_) of Que in Exo-Que was higher to 7.5-fold as compared to free Que.

In order to investigate whether administration of Exo-Que was safe and suitable for long-term therapy, we evaluated the toxicity of Que and Exo-Que on main organs by observing their morphology using H&E staining. The results (Figure 4(B)) showed that after treated with Exo-Que, the morphologies of main organs including heart, liver, spleen, lung and kidney were normal and appeared no difference as compared with saline-treated group (sham group). This indicated that all preparations including Que and Exo-Que were actually safe and had no overall systematic toxicity *in vivo*.

### Que enhanced its accumulation in brain region with the help of Exo

**Figure 5.**
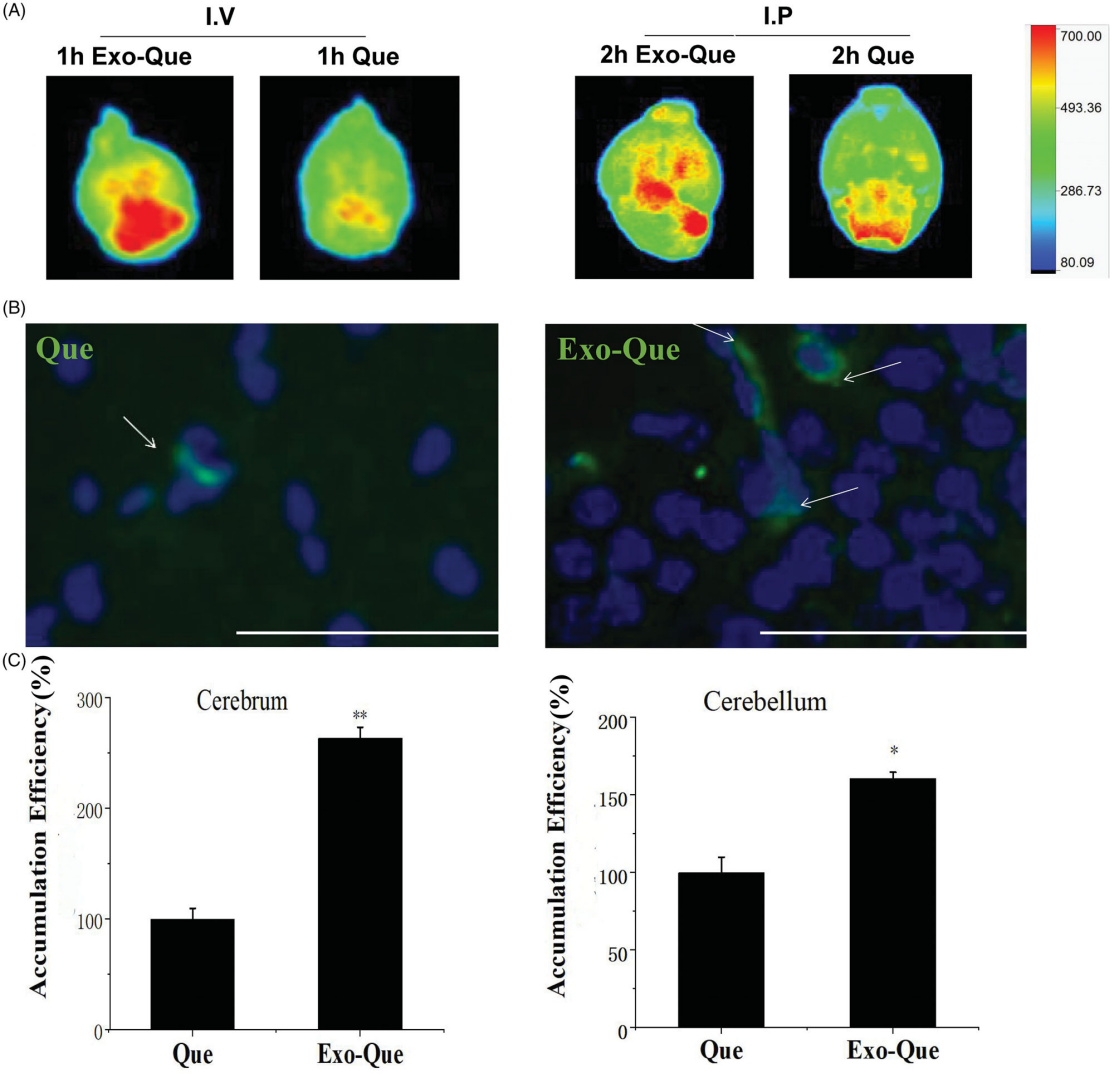
The brain targeting of Exo-Que *in vivo*. (A) Representative fluorescence images of C57BL/6 mice brains treated with Que and Exo-Que via intravenous injection and peritoneal injection. (B) Representative fluorescent images of Que and Exo-Que (white arrows) at hippocampus of C57BL/6 mice after 24 h of i.p. administration of Que and Exo-Que. The scale bar is 100 μm and applies to all figure parts. (C) The content determination of Que in cerebrum and cerebellum of rats treated with single i.v. administration of Que and Exo-Que. The accumulation efficiency of Que was set as 100%. Data are expressed as means ± SD (*n* = 3), *p < 0.05, **p < 0.01.

In order to find out the brain-targeting efficacy of Que, Que and Exo-Que were given to mice by intravenous injection (I.V.) and peritoneal injection (I.P.), the brain of mice were dissected and an IVIS Spectrum imaging system (PerkinElmer, Waltham, MA, USA) was used to capture Que emitted fluorescence images (excitation at 436 nm and emission at 486 nm) in brain. It showed that in Figure 5(A) Exo-Que significantly enhanced accumulation of Que in brain and highly enhanced fluorescence of Que was observable in brain in Exo-Que-treated group in contrast with weakening fluorescent intensity of Que in Que-treated group. Furthermore, green color fluorescence of Que was well localized in hippocampus of brain, showing in Figure 5(B). We collected rats brain at 1 h after i.v. administration of Que and Exo-Que, and the concentrations of Que were detected by HPLC. As expected with the previous results, Exo increased Que concentration in brain up to 2.5-fold in cerebrum and 1.5-fold in cerebellum compared with free Que (Figure 5(C)). These data supported that Exo-Que showed enhanced brain targeting and more exosomal Que was delivered into hippocampus of brain as compared with free Que.

### Exo-Que rescued the cognitive dysfunction of OA-induced AD mice

**Figure 6.**
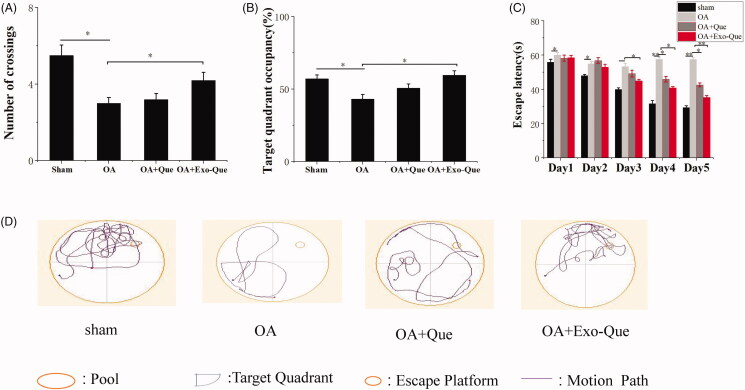
Exo-Que ameliorated the cognitive deficiency in OA-treated AD mice. (A) The platform crossing number in the spatial probe test. Data are expressed as mean ± SD (*n* = 3). * *p* < 0.05. (B) Relative time percentage spent on the target quadrant in a probe trial. Data are expressed as mean ± SD (*n* = 3). * *p* < 0.05. (C) Escape latencies analysis. Data are expressed as mean ± SD (*n* = 3). * *p* < 0.05, ** *p* < 0.01. (D) Representative path tracings of different groups.

We investigated whether Exo-Que could ameliorate the OA-induced cognitive dysfunction in mice. The effect of Exo-Que on attenuating learning and memory deficits was also investigated by Morris water maze test. It showed in Figure 6 that OAinduced cognitive dysfunction in mice which was manifested as the lower target quadrant occupancy and less numbers of crossing as compared to that sham group. After administrated with Que, there was no significant difference (*p* > 0.05) in escape latencies and target quadrant occupancy compared with the OA group. On the contrary, Exo-Que significantly enhanced memory and spatial learning performance. Exo-Que showed the increased crossing numbers, highest target quadrant occupation and the reduced escape latencies among all groups. In summary, it supported therapeutic efficacy of Exo-Que as a neuroprotective agent for improving cognition in OA-induced AD mice.

### Exo-Que exerted neuroprotective outcome in OA-induced AD mice by reducing apoptosis of neuron cells

**Figure 7.**
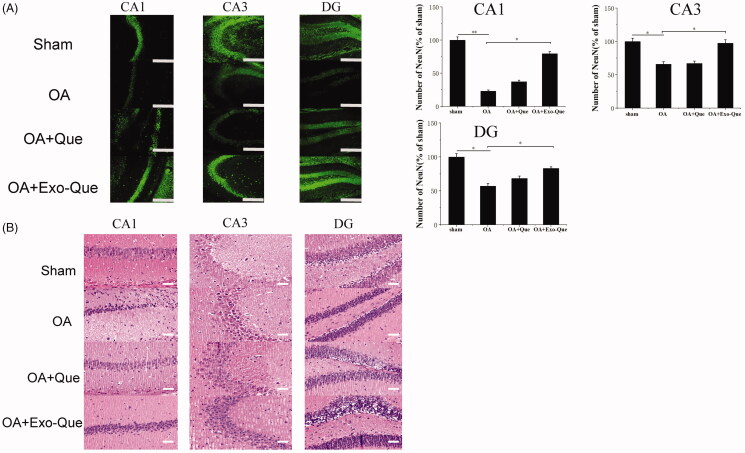
(A) Representative immunofluorescence staining for neuron cell in CA1, CA3, and DG sector of the hippocampus via peritoneal injection of saline, Que and Exo-Que. Neun antibodies were used to stain neuron cells in hippocampus of brain. The scale bar is 250 μm and applies to all figure parts. Data are expressed as mean ± SD (*n* = 3). * *p* < 0.05, ** *p* < 0.01. (B) H&E staining of hippocampal sections treated with saline, Que and Exo-Que. Scale bar: 50 μm.

In view of the improved learning and memory ability of Exo-Que, we further investigated whether exosomal Que-induced neuroprotection via reversing the apoptosis of neuron cells. The number of neurons in hippocampus was observed and counted by immunofluorescence staining and H&E staining. It showed in Figure 7 that OA reduced the number of NeuN-positive neuron cells and induced histopathological damage by showing swollen neuronal bodies, shrinkage of nuclei and neuron rarefaction in the hippocampus. As expected, compared with OA and OA + Que group, Exo-Que significantly increased the number of NeuN-positive neuron cells in hippocampal CA1 region, CA3 region and DG region. H&E staining results also showed that compared with OA and OA + Que group, the neuronal layers in the hippocampus showed integrity and orderliness maintaining intact neuronal cells structure with clear line and abundant cytoplasm in OA + Exo-Que group. All results showed that Exo-Que indeed further induced neuroprotection and suppressed the apoptosis of neurons in hippocampus.

### Naïve Exo exerted a synergistic neuroprotection on rescuing memory deficits in OA mice

**Figure 8.**
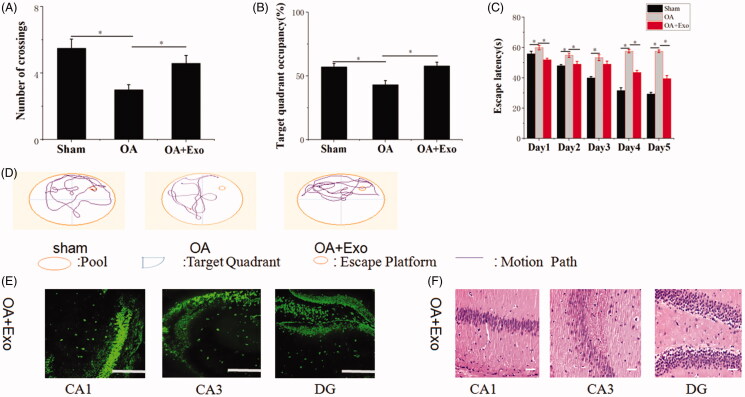
Neuroprotective role of naïve Exo on OA mice. (A) The platform crossing number in the spatial probe test. Data are expressed as mean ± SD (*n* = 3). *p < 0.05. (B) Relative time percentage spent on the target quadrant in a probe trial. Data are expressed as mean ± SD (*n* = 3). *p < 0.05. (C) Escape latencies analysis. Data are expressed as mean ± SD (*n* = 3). *p < 0.05. (D) Representative path tracings of different groups. (E) Representative immunofluorescence staining for neuron cell in CA1, CA3 and DG sector of the hippocampus via peritoneal injection of Exo. Neun antibodies were used to stain neuron cells in hippocampus of brain. The scale bar is 250 μm and applies to all figure parts. (F) H&E staining of hippocampal sections treated with Exo. Scale bar: 50 μm.

In our previous work, we had revealed naïve Exo protected against cerebral diseases. Hence, we asked whether naïve Exo participated in modulating recognition and induced neuroprotection. The results (Figure 8) revealed that compared with OA group, Exo treatment reduced OA-induced death of neuron cells and more NeuN-positive cells maintaining intact cells structure were observed in hippocampal CA1 region, CA3 region and DG region. Furthermore, OA mice treated with Exo showed spatially oriented swimming behavior, improved memory and spatial learning ability by showing the shorter escape latencies, higher target quadrant percentage and increased numbers of crossing in Morris water maze test. However, neuroprotective role of naïve Exo was limited relative to Exo-Que by lower percentage of occupancy in target quadrant and longer escape latency time. It confirmed that naïve Exo contributed to synergistic neuroprotection and attenuated OA-induced cognitive decline by regulating neuron cells.

### Exo-Que inhibited formation of insoluble neurofibrillary tangles (NFTs) by reducing CDK5 medicated phosphorylation of tau protein by OA *in vivo*

**Figure 9.**
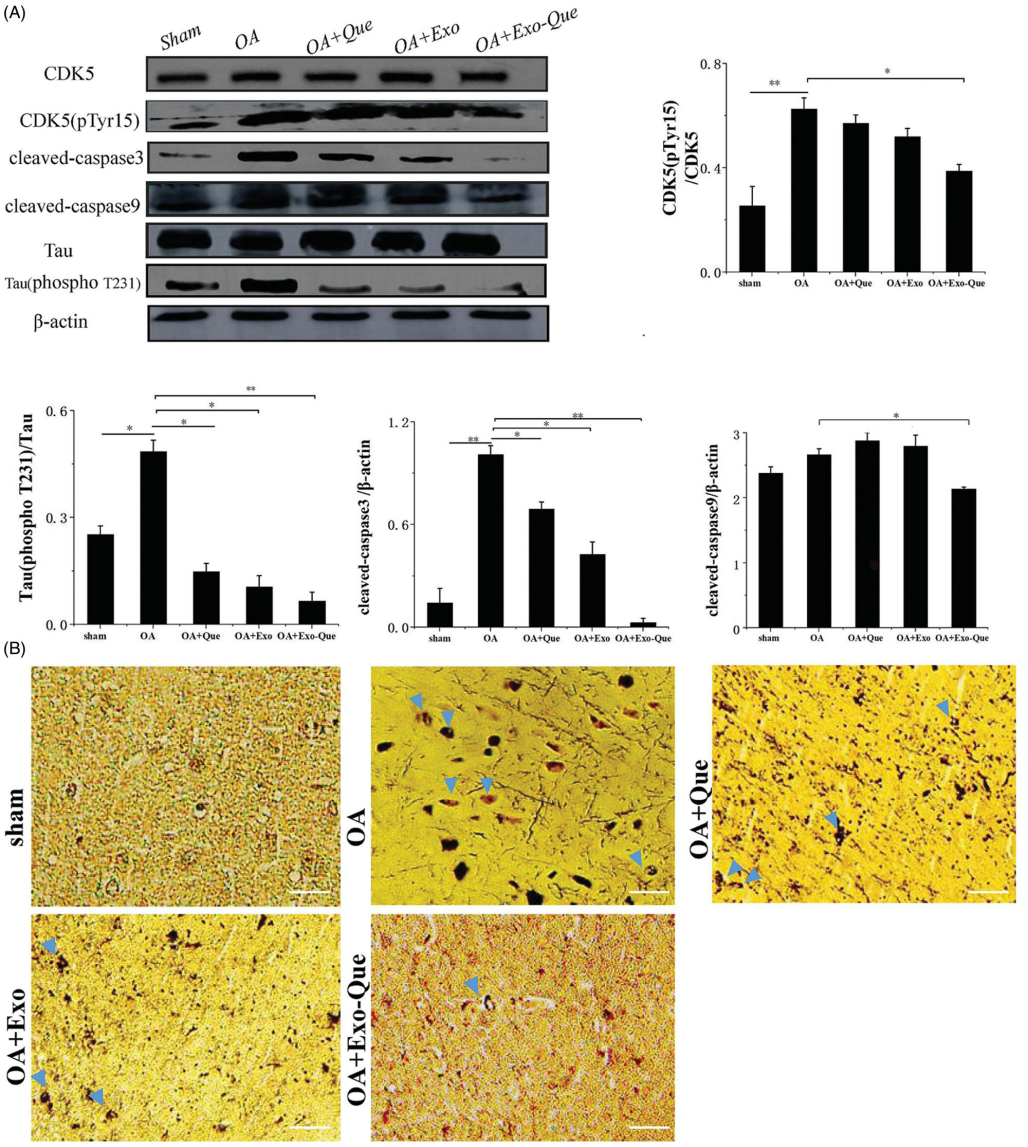
Analysis of Exo-Que on neuroprotective effects in OA-induced AD mice through inhibiting CDK5-mediated phosphorylation of Tau and reducing formation of insoluble neurofibrillary tangles. (A) Western blot and quantitative analysis of the expression levels of CDK5 phosphor-CDK5 (pTyr15), Tau, phosphor-Tau (T231), cleaved caspase3 and cleaved caspase9 in sham, OA, OA + Que, OA + Exo and OA + Exo-Que groups. Data are expressed as mean ± SD (*n* = 3), * *p* < 0.05, ** *p* < 0.01. (B) The images of NFTs (blue arrows) on brain section in sham, OA, OA + Que, OA + Exo and OA + Exo-Que groups. Scale bar: 50 μm.

As it is known that phosphorylated Tau-induced NFTs cause loosing connectivity of the affected neurons and impaire the normal neuron function, we focused on the effect of Exo-Que on inhibiting the over-phosphorylation of Tau protein induced by OA and reducing the production of NFTs. As shown in Figure 9, it revealed that compared with the sham group, the number of NFTs was significantly increased and the expression levels of phosphorylated CDK5 and Tau phosphorylation were enhanced in OA mice. Furthermore, the apoptosis related proteins such as cleaved caspase 9 and cleaved caspase 3 were significantly up-regulated. It indicated that OA administration induced the hyperphosphorylation of CDK5 and Tau, thus accelerating massive accumulation of NFTs and aggravating neurological deficits. In the contrast, Exo-Que reduced the production of NFTs and inhibited the phosphorylation of CDK5 and Tau. Furthermore, the expressions of cleaved caspase 9 and cleaved caspase 3 in Exo-Que-treated group were significantly down-regulated. This result inferred that Exo-Que inhibited formation of NFTs by reducing CDK5-mediated phosphorylation of Tau protein by OA *in vivo*, thus contributing to its enhanced neuroprotection and functional improvement.

## Discussion

AD is a chronic neurodegenerative disorder characterized by progressive cognitive impairment and memory loss. Despite selected clinical medications can temporarily alleviate patients’ memory deficit and improve their life quality, there is still no effective curing strategy for fighting against AD. Especially, in the past few years, a number of compounds which inhibited the production and aggregation of amyloid-β (Aβ) failed to pass the final clinical trial. In view of the repeated failures of new clinically Aβ targeted AD drugs, more attention switched to finding potential drugs targeting the hyperphosphorylation of tau protein to alleviate cognitive dysfunction in AD therapy.

Que as a plant-derived ‘flavonoid’ found in fruits and vegetables shows antioxidant, anti-inflammatory, antitumor, and antiviral properties, thus inducing potential beneficial effects on human disease therapy, It is reported that Que can target hippocampal neurons and prevent tau-hyperphosphorylated neurodegeneration by possessing an significant inhibition on tau-fibril aggregation. However, it has also inherited some pitiable shortcomings such as short half-life, BBB crossing difficulty and low bioavailability, thus limiting the effective use of Que in the clinical treatment of AD.

In this study, we chose plasma exosomes (Exo) as a therapeutic cargo carrier to deliver Que into brain. Unlike few production of cell derived exosomes with longer time span, a large amount of Exo could be easily produced in a short time by ultracentrifugation of a plenty supply of blood. Furthermore, our unpublished data suggested that Exo could across BBB and induce enhanced brain migration of drug. We found that expression levels of endothelial Toll-like receptor 4 (TLR4) was highly expressed in brain and Exo was enriched with heat shock protein 70 (HSP70). Exo might enhance the BBB crossing by specific active targeting between Exo-inherited HSP70 and endothelial TLR4 in brain. We also found that Exo contributed to a synergistic action on reducing apoptosis of neuron cells and attenuating cognitive decline in OAinduced AD mice by downregulating hyperphosphorylation of Tau protein. Herein, we fabricated Que-encapsulated Exo for enhancing its bioavailability and brain targeting ability. The results showed that Exo-Que presented homogeneous and spherical vesicles. No significant toxicity of Exo-Que was observed in animals by histopathological analysis, suggesting that Exo-Que was biocompatible and safe. Compared with free Que, Exo-Que changed Que pharmacokinetic profiles and depended on Exo brain targeting properties to migrate more Que into brain. Furthermore, Exo-Que significantly attenuated OA-induced neurodegeneration in mice by reducing the apoptosis of neuron cells and improving memory and spatial learning ability as compared to Que-treated group. We also explained Exo-Que-induced neuroprotection from molecular signaling pathway by western blot. It was found that Exo-Que significantly inhibited the activity of CDK5 and decreased tau protein hyperphosphorylation, thereby reducing the formation of NFTs. Exo-Que also showed the better anti-apoptosis effects by inducing the lower expressions of cleaved caspase9 and cleaved caspase3.

## Conclusion

In conclusion, Exo-Que enlarged neuroprotective effects of Que through enhancing the bioavailability of Que and promoting its brain targeting. Furthermore, it significantly rescued memory deficits in OA-induced AD mice by inhibiting CDK5-mediated phosphorylation of Tau and reducing formation of NFTs. These results supported the idea that Exo-Que as a potent inhibitor of tau protein aggregation was more efficacious for improving cognitive function in AD mice and regarded as a potential therapeutic strategy for AD therapy.
